# Three new species of *Epicephala* Meyrick (Lepidoptera, Gracillariidae) associated with *Phyllanthus microcarpus* (Benth.) (Phyllanthaceae)

**DOI:** 10.3897/zookeys.484.8696

**Published:** 2015-03-05

**Authors:** Houhun Li, Xiaofei Yang

**Affiliations:** 1 College of Life Sciences, Nankai University, Tianjin 300071, P. R. China

**Keywords:** Lepidoptera, Gracillariidae, Phyllanthaceae, *Epicephala*, *Phyllanthus*, new species, China

## Abstract

Three new species of *Epicephala* Meyrick, 1880 are described based on specimens reared from fruits of *Phyllanthus microcarpus* (Benth.): *Epicephala microcarpa* **sp. n.** and *Epicephala laeviclada* **sp. n.** from Guangxi and Hainan, and *Epicephala tertiaria* **sp. n.** from Guangdong and Guangxi. Photographs of adults and illustrations of genital structures are provided.

## Introduction

The genus *Epicephala* Meyrick, 1880 of the moth family Gracillariidae has been reported to have close coevolutionary relationships with the genera *Glochidion*, *Phyllanthus* and *Breynia* of the plant family Phyllanthaceae. *Epicephala* currently consists of 46 described species worldwide, mainly distributed in the Old World (Vári 1961; Kuznetzov 1979; Nielsen et al. 1996; De Prins and De Prins 2005, 2011; Zhang et al. 2012). In China, nine species have been recorded prior to this study (Zhang et al. 2012).

**Figures 1–5.**
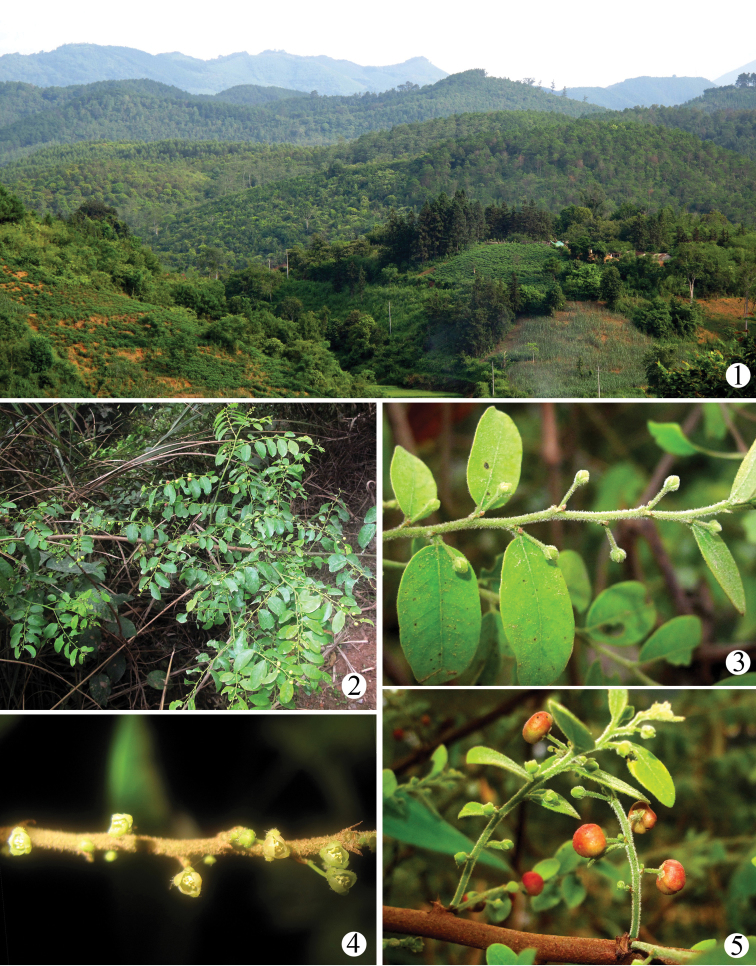
Habitats of *Phyllanthus microcarpus* (Benth.), the host plant of *Epicephala* species in Shaoping Forestry Centre, Pingxiang, Guangxi. **1** general habitat; **2–5** morphological features: **2** an individual tree **3** female flowers and leaves **4** male flowers **5** female flowers and fruits.

The present paper describes three new species based on specimens reared from the host-plant, *Phyllanthus microcarpus* (Benth.) (Figs 1–5) from Guangxi Zhuang Autonomous Region, Hainan and Guangdong provinces, while the authors were studying their biology and coevolution with the host-plant (to be reported upon in different papers). *Phyllanthus microcarpus* was previously a synonym of *Phyllanthus reticulatus* Poir. until Luo et al. (2011) showed they are two different species, with differences in vegetative and floral characteristics, and different habitats and distribution.

## Material and methods

The field study was conducted from 2011 to 2013 in Pingxiang, Guangxi Zhuang Autonomous Region, and from 2009 to 2014 in several nature reserves in Hainan Province, China. Specimens examined in this study were collected or reared from fruits of *Phyllanthus microcarpus* (Benth.). Genitalia dissection and mounting methods follow Li and Zheng (1996). Photos of the host-plants were taken in the field using a Canon PowerShot G10 digital camera. Photos of adult specimens were taken with a Leica M250A stereo microscope, and illustrations of the genitalia were prepared by using a Leica DM750 microscope, and refined in Photoshop®CS4 software.

The type specimens are deposited in the Insect Collection, College of Life Sciences, Nankai University, Tianjin, China and some paratypes are deposited in the Department of Entomology, Natural History Museum, London, UK (BMNH).

## Description of new species

### Epicephala microcarpa

Taxon classificationAnimaliaLepidopteraGracillariidae

Li sp. n.

http://zoobank.org/F9726A27-9218-4780-BA70-36BFE564B432

Figs 6 , 9 , 12

#### Material examined.

237 males and 206 females, including all their genitalia preparations.

Holotype ♂ – **CHINA:** **Hainan Province:** Diaoluoshan, 18.xii.2012, reared from fruit of *Phyllanthus microcarpus* Poir. by Zhibo Wang, genitalia slide no. WZB14371.

Paratypes – **CHINA:** **Hainan Province:** 3♂, 1♀, Nanxi Forestry Station, Diaoluoshan, Lingshui County, 300 m, 9–15.viii.2008, under light trap, leg. Bingbing Hu and Li Zhang; 4♂, 4♀, Diaoluoshan, 12–29.iv.2008, 11.xi-10.xii.2009, reared from fruits of *Phyllanthus microcarpus* by Bingbing Hu, 12♂, 14♀, 18.xii.2012, reared from fruits of *Phyllanthus microcarpus* by Zhibo Wang; 11♂, 11♀, Tropical Botanical Garden, Danzhou, 30.xi-28.xii.2009, reared from fruits of *Phyllanthus microcarpus* by Bingbing Hu; 1♂, 3♀, Yinggeling Mountain Nature Reserves (19°01'N, 109°33'E), 450 m, 8–20.vi.2010, reared from *Phyllanthus microcarpus* by Bingbing Hu; 3♂, 1♀, Jianfenling, 24.vi.2010, leg. Bingbing Hu. **Guangxi Zhuang Autonomous Region:** 171♂, 203♀, Shaoping Forestry Centre (22°05'N, 106°54'E), 200 m, Pingxiang, 22.vii–12.viii.2011, 6.iv–28.vii.2012, 27.iii–22.vii.2013, reared from fruits of *Phyllanthus microcarpus* by Xiaofei Yang (2♂, 2♀, deposited in BMNH). **INDIA:** 1♂, label 1: Surat, Bombay, RM. 24.1.[19]29; label 2: *Epicephala vermiformis*, 1/2 Meyr., E. Meyrick det., in Meyrick Coll.; label 3: Meyrick Coll., B. M. 1938–390; label 4: B. M. Genitalia slide No. 32328, dissected by Houhun Li, deposited in the Natural History Museum, London (BMNH).

#### Diagnosis.

This species is similar to *Epicephala exetastis* Meyrick, 1908 both in appearance by having similar densely compacted markings and in the genital structures. It can be separated from the latter in the female by the broad cone-shaped ovipositor, the inconspicuous lamella postvaginalis, the expanded antrum and ductus bursae, and the broad signa. In *Epicephala exetastis* Meyrick, the ovipositor is slender, the lamella postvaginalis is conspicuous, the antrum and ductus bursae are narrow, and the signa are narrow in the female.

#### Description.

**Figures 6–8.**
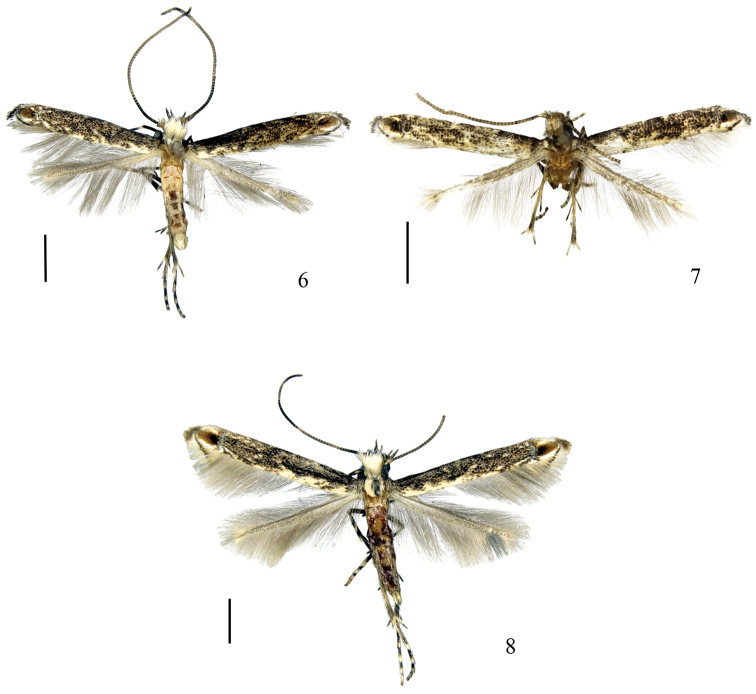
Adults of *Epicephala* spp. **6** *Epicephala microcarpa* sp. n., paratype ♂ **7** *Epicephala laeviclada* sp. n., holotype ♂ **8** *Epicephala tertiaria* sp. n., paratype ♀ (Scale bars = 1.0 mm).

Adult (Fig. 6). Forewing expanse 5.0−7.5 mm. Head white to pale yellowish brown, lateral sides with long black scales. Labial palpus black, inner surface of second and distal portion of third segments mixed with white scales. Antenna dark brown, with narrow greyish white rings, more distinct on dorsal surface. Thorax white. Tegula with basal half brown, distal half greyish white. Forewing greyish brown to dark brown, markings dense and compact; three pairs of white striae from both costal and dorsal 2/3, 1/2 and 3/4 extending obliquely outward to middle and end of cell as well as outside of cell, dorsal striae broader and clearer than costal striae; basal 1/6 of dorsum with broad white band; a narrow silvery-white fascia with metallic reflection from costal 5/6 to dorsum, arched outward medially; distal 1/6 ochre brown, with a central black dot edged by a short white streak or a dot near costa, with a white band along dorsum; cilia greyish white except black at base and apex, adjacent white from costal margin along termen to tornus, then grey along dorsal margin. Hindwing and cilia greyish brown. Abdomen dark brown.

**Figures 9–11.**
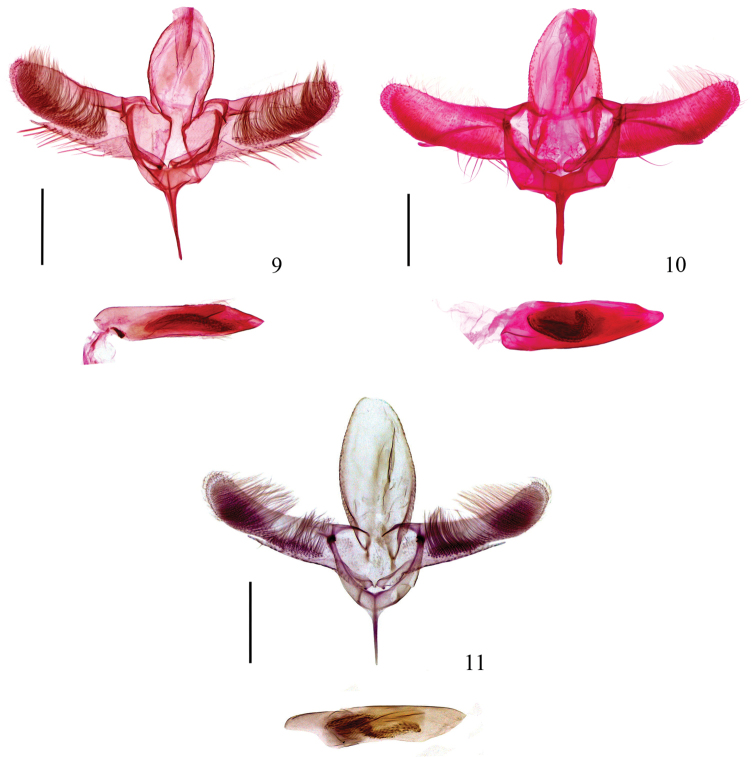
Male genitalia of *Epicephala* spp. **9** *Epicephala microcarpa* sp. n., paratype, genitalia slide No. WZB14371 **10** *Epicephala laeviclada* sp. n., paratype, genitalia slide No. YXF14282 **11** *Epicephala tertiaria* sp. n., paratype, genitalia slide No. ZJ10021 (Scale bars = 0.2 mm).

**Male genitalia** (Fig. 9). Tegumen broadly elliptical, lateral sides narrow and sclerotized. Valva rectangular, longer than tegumen, nearly parallel dorso-ventrally, apex obliquely rounded, with long dense setae ventrally. Sacculus narrowed, elongate triangular, approximately 4/5 length of valva, tapered to sharp or truncate apex distally; densely with long setae ventrally. Transtilla slender, S-shaped, curved downward distally, acute apically. Vinculum broad, nearly U-shaped; saccus slender, nearly the same length as vinculum, apex rounded. Phallus straight, approximately 3/4 length of valva; cornuti formed by dense spinules grouped into two bundles.

**Figures 12–14.**
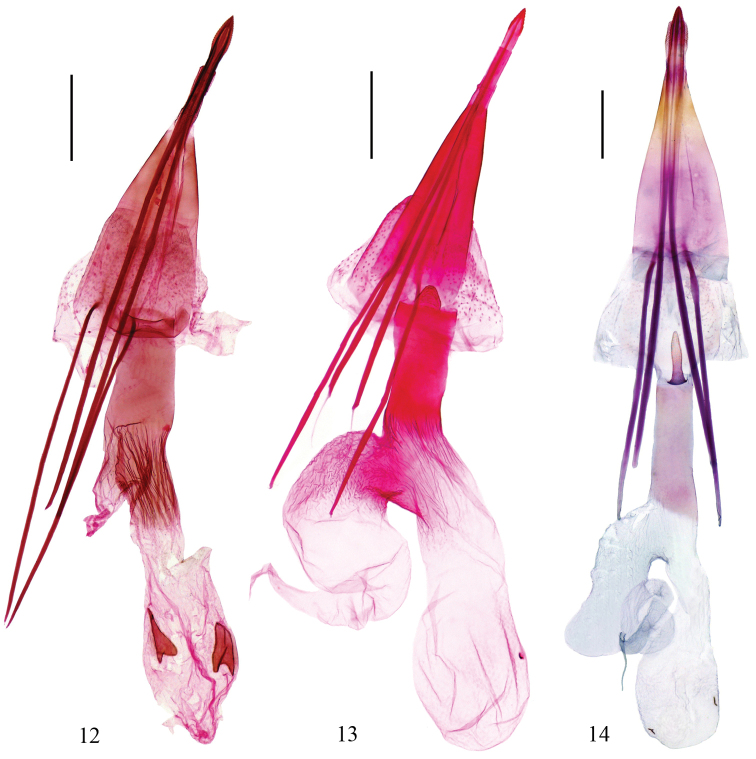
Female genitalia of *Epicephala* spp. **12** *Epicephala microcarpa* sp. n., paratype, genitalia slide No. YXF14026 **13** *Epicephala laeviclada* sp. n., paratype, genitalia slide No. YXF14060 **14** *Epicephala tertiaria* sp. n., paratype, genitalia slide No. ZJ10028 (Scales = 0.2 mm).

**Female genitalia** (Fig. 12). Ovipositor broad, cone-shaped, constricted basally, dentate laterally, acute apically. Apophysis posterioris strong, 1.2 times longer than apophysis anterioris. Lamella postvaginalis broad and very short, unconspicuous. Antrum thick, strongly sclerotized, nearly as long as 8th abdominal segment. Ductus bursae broad, slightly longer than antrum, basal 2/3 sclerotized with wide longitudinal pleats; ductus seminalis expanded, arising from base of ductus bursae. Corpus bursae oval, shorter than ductus bursae, medially with pair of large signa, apex of signum with two teeth.

#### Host-plant.

Phyllanthaceae: *Phyllanthus microcarpus* (Benth.). The larva feeds on seeds in the fruit.

#### Distribution.

China (Guangxi and Hainan), India (Bombay).

#### Etymology.

This new species is named after its host-plant *Phyllanthus microcarpus* (Benth.).

#### Remarks.

One specimen of the new species collected in India was determinated as *Epicephala vermiformis* Meyrick, 1936 by Meyrick himself. However, this specimen is quite different from the two Indonesian syntypes of *Epicephala vermiformis* by having a distinctly narrower forewing (Natural History Museum, London, examined). Moreover, the host-plant of *Epicephala vermiformis* is *Cajanus cajan* (L.) (Fabaceae) (De Prins and De Prins 2014), while all species in the genus are host-specific.

### Epicephala laeviclada

Taxon classificationAnimaliaLepidopteraGracillariidae

Li sp. n.

http://zoobank.org/CE54CF03-7092-4998-834D-5AF25EDDC43C

Figs 7 , 10 , 13

#### Material examined.

10 males and 5 females, including all their genitalia preparations.

Holotype ♂ – **CHINA:** **Guangxi Zhuang Autonomous Region:** Shaoping Forestry Centre (22°05'N, 106°54'E), 200 m, Pingxiang, 20.vi.2012, reared from fruit of *Phyllanthus microcarpus* (former identification Phyllanthus reticulatus var. glaber) by Xiaofei Yang, genitalia slide no. YXF14198.

Paratypes – **CHINA:** **Guangxi Zhuang Autonomous Region:** 9♂, 4♀, same locality and host-plant as holotype, 26.vii.2011, 26.iv–24.vi.2012, 27.iii–14.iv.2013 collected under light or reared from fruits of host-plant. **Hainan Province:** 1♀, Tropical Botanical Garden, Danzhou, 30.xi.2009, reared from fruits of *Phyllanthus microcarpus* (Benth.) by Bingbing Hu.

#### Diagnosis.

This species is similar to *Epicephala microcarpa* sp. n. in appearance, but can be separated from the latter by the compacted sacculus with bluntly rounded apex that connects with a ridge in the inner surface of the valva, the stout bullet-like phallus with cornuti composed of spinules that are grouped into one bundle in the male; the cone-shaped lamella postvaginalis is conspicuous, and the corpus bursae with only one small signum in the female. In *Epicephala microcarpa* sp. n., the sacculus is narrower and longer, and its apex is usually sharp and lacks a sclerotized ridge in the inner surface of the valva, and the straight phallus has cornuti composed of spinules that are grouped into two bundles in the male; the broad and very short lamella postvaginalis is unconspicuous, and the corpus bursae has a pair of large signa in the female.

#### Description.

Adult (Fig. 7). Forewing expanse 5.0−7.5 mm. Head white to greyish brown, lateral sides with long black scales. Labial palpus black, inner surface greyish white to black, basal 1/3 of second and both ends of third segments greyish white. Antenna dark brown, scape with long and narrow scales, flagellum with narrow greyish rings. Thorax white to greyish brown. Tegula brown, apically greyish white. Forewing brown to dark brown; three white striae from costal 1/4, 1/3 and 2/5 extending obliquely outward to 1/3 width of forewing; dorsum with broad white band along basal 1/3, serrated on upper edge, distally with a stria extending obliquely outward to middle of cell, with a small triangular white spot and an obliquely outward stria at middle and before 5/6, respectively; a narrow silvery-white fascia with metallic reflection from costal 5/6 to dorsum; distal 1/6 ochreous, with a central black spot edged by a white dot near costa and a white band along dorsum; cilia greyish white except black at basal margin and apex. Hindwing and cilia greyish brown. Abdomen dark brown.

**Male genitalia** (Fig. 10). Tegumen elongate elliptical, lateral sides narrow and sclerotized. Valva rectangular, somewhat longer than tegumen, nearly parallel dorso-ventrally, costal margin gently curved, apex rounded. Sacculus narrowed, compact, elongate triangular, approximately 2/3 length of valva, apex bluntly rounded and connected with sclerotized ridge obliquely arched to base of vinculum; sparsely with long setae ventrally. Transtilla S-shaped, stout basally, curved downward distally, acute apically. Vinculum short and broad, somewhat rectangular; saccus slender, nearly the same length as vinculum, apex bluntly rounded. Phallus stout, bullet-like, approximately 3/4 length of valva; cornuti composed of dense spinules grouped into a bundle.

**Female genitalia** (Fig. 13). Ovipositor broad, cone-shaped, dentate laterally, acute apically. Apophysis posterioris strong, 1.2 times longer than apophysis anterioris. Lamella postvaginalis situated at base of antrum medially, short cone-shaped, approximately 2/5 width of antrum, same length with width. Antrum thick, heavily sclerotized, slightly longer than 8th abdominal segment. Ductus bursae membranous, broadly expanded, as long as antrum; ductus seminalis expanded, arising from base of ductus bursae. Corpus bursae oval, shorter than ductus bursae, medially with a small semilunar signum.

#### Host-plant.

Phyllanthaceae: *Phyllanthus microcarpus* (Benth.). The larva feeds on seeds in the fruit.

#### Distribution.

China (Guangxi and Hainan).

#### Etymology.

The specific name is derived from the Latin *laevis* (smooth) and *cladus* (branch), in reference to individuals of the host-plant, *Phyllanthus microcarpus* (Benth.), having glabrous branches.

#### Remarks.

The host-plant, *Phyllanthus microcarpus* (Benth.), has glabrous and pubescent forms that were formerly identified as the varieties Phyllanthus reticulatus var. glaber (glabrous) and Phyllanthus reticulatus var. reticulatus (pubescent). However, *Phyllanthus reticulatus* also has such forms, and other characters are needed to separate the two plant species. The larva of *Epicephala laeviclada* sp. n. has only been found on the glabrous plants.

### Epicephala tertiaria

Taxon classificationAnimaliaLepidopteraGracillariidae

Li sp. n.

http://zoobank.org/ECB0B192-9905-44D7-BE46-520BBB219537

Figs 8 , 11 , 14

#### Material examined.

21 males and 14 females, including all their genitalia preparations.

Holotype ♂ – **CHINA:** **Guangdong Province:** South China Botanical Garden, Chinese Academy of Sciences, Guangzhou, (23°11'N, 113°22'E), 210 m, 22.ii.2006, reared from fruit of *Phyllanthus microcarpus* (former identification Phyllanthus reticulatus var. glaber) by Houhun Li, genitalia slide no. YXF14039.

Paratypes – **CHINA:** **Guangdong Province:** 11♂, 7♀, same data as holotype; 7♂, 4♀, same locality and host-plant, vii-viii.2006, collected mature larvae by Shixiao Luo and reared to adults by Houhun Li. **Guangxi Zhuang Autonomous Region:** 2♀, Shaoping Forestry Centre (22°05'N, 106°54'E), 200 m, Pingxiang, 24, 29.vi.2012, reared from fruit of *Phyllanthus microcarpus* (former identification Phyllanthus reticulatus var. reticulatus) by Xiaofei Yang; 2♂, same locality, 27.iii, 10.iv.2013, reared from fruit of *Phyllanthus microcarpus* by Xiaofei Yang, whether the plants are glabrous or pubescent was not recorded.

#### Diagnosis.

This species is similar to *Epicephala microcarpa* sp. n. in both appearance and genitalia, but can be separated from the latter by distal 1/6 of the forewing having a broad white band along costa; the narrower valva as long as the tegumen and rounded at apex, and the narrower and shorter sacculus approximately 2/3 length of the valva in the male; the ovipositor not constricted basally, the lamella postvaginalis digitated, the ductus bursae membranous, and the smaller corpus bursae with very minute signa in the female. In *Epicephala microcarpa* sp. n., the forewing has a short white streak or a dot near costa in distal 1/6; the valva is broader and longer than the tegumen and its apex is oblique, the sacculus is somewhat broader and approximately 4/5 length of the valva in the male; the ovipositor is constricted at base, the lamella postvaginalis is unconspicuous, basal 2/3 of the ductus bursae is sclerotized and densely covered with longitudinal wide pleats, and the signa are large in the female.

#### Description.

Adult (Fig. 8). Forewing expanse 6.0−8.5 mm. Head cream white, with dark brown laterally. Labial palpus black, inner surface and outer ventral margin of second segment white, inner surface of third segment white to greyish brown. Antenna dark brown, with narrow greyish white rings. Thorax white. Tegula and forewing brown to dark brown; forewing with three pairs of white striae from both costal and dorsal 1/4, 2/3 and 3/4 extending obliquely outward to middle and end of cell as well as outside of cell respectively, costal striae narrow, inconsecutive and usually indistinct, dorsal striae broad and clear, latter two striae inconsecutive; dorsum with a broad white band along basal 1/3; a narrow silvery-white fascia bearing bluish metallic reflection from costal 5/6 to dorsum, arched outward medially; distal 1/6 ochreous, with a central black dot near fascia at 5/6, with broad white band along costa and dorsum; cilia along termen to tornus pale grey except black at base and ochre brown at apex. Hindwing and cilia pale grey. Abdomen greyish brown.

**Male genitalia** (Fig. 11). Tegumen broadly elliptical, lateral sides narrow and sclerotized. Valva narrowed, rectangular, as long as tegumen, slightly narrowed medially, gently curved upward, apex rounded, with long dense setae ventrally. Sacculus narrowed, approximately 2/3 length of valva, tapered to sharp apex. Transtilla slender, curved downward, acute apically. Vinculum broad, nearly U-shaped; saccus slender, shorter than vinculum, apex acute. Phallus broad, straight, approximately 3/4 length of valva; cornuti composed of dense spinules.

**Female genitalia** (Fig. 14). Ovipositor broad, cone-shaped, dentate laterally, acute apically. Apophysis posterioris strong, 1.2 times longer than apophysis anterioris. Lamella postvaginalis digitated, arising from base of antrum medially, 1/4 length of apophysis anterioris, apex rounded. Antrum developed, cylindrical, straight, longer than 8th abdominal segment. Ductus bursae narrow, membranous, shorter than antrum; ductus seminalis expanded, broader than ductus bursae, arising from base of ductus bursae. Corpus bursae oval, small, as long as ductus bursae; paired signa placed anteriorly, small, short linear.

#### Host-plant.

Phyllanthaceae: *Phyllanthus microcarpus* (Benth.). The larva feeds on seeds in the fruit.

#### Distribution.

China (Guangdong and Guangxi).

#### Etymology.

The specific name is derived from the Latin *tertiarius* (third), indicating that this is the third species reared from the host-plant *Phyllanthus microcarpus* (Benth.).

#### Remarks.

The larvae were reared from glabrous individuals of *Phyllanthus microcarpus* in Guangzhou, Guangdong Province, and from pubescent individuals of *Phyllanthus microcarpus* in Pingxiang, Guangxi Zhuang Autonomous Region. Both glabrous and pubescent forms are now treated as one species (Luo et al. 2011). This interesting phenomenon may have some significance in the coevolution between the *Epicephala* moths and the Phyllanthaceae plants.

## Supplementary Material

XML Treatment for Epicephala microcarpa

XML Treatment for Epicephala laeviclada

XML Treatment for Epicephala tertiaria
